# Oral administration of *Lactiplantibacillus plantarum* 6.2 mitigates acute lung injury via immunostaining of the TLR4/NF-κB pathway and reduction of IL-17 in lung tissue

**DOI:** 10.1007/s10123-026-00853-w

**Published:** 2026-06-19

**Authors:** Giovanna Pinheiro Martins, João Pedro S. Cândido, Rubens da Silva Araújo, Jannerson Cesar Xavier de Pontes, Rachel Passos Rezende, Carla Cristina Romano, Adailson de Souza Assunção, Daniele Idalino Janebro, Janeusa Trindade de Souto, Wallace Felipe Blohem Pessoa, Adriano Francisco Alves

**Affiliations:** 1 Postgraduate Program in Natural and Synthetic Products Bioactive/Health Sciences Center, Federal University of Paraiba, Joao Pessoa, 58051-900 Brazil; 2 Biomedical Sciences Department, Federal University of Paraiba, Joao Pessoa, Brazil; 3 Postgraduate program in Dentistry, Federal University of Paraiba, Joao Pessoa, Brazil; 4 Department of Biological Sciences, State University of Santa Cruz – UESC, Ilhéus, Bahia Brazil; 5 Center of Biosciences, Federal University of Rio Grande do Norte – UFRN, Natal, Brasil

**Keywords:** ALI, LPS, Lactobacilli, Probiotics, Inflammation

## Abstract

**Background:**

Acute lung injury (ALI) is an inflammatory condition that compromises the lungs and is associated with substantial morbidity and mortality. In the absence of effective therapeutic options, the search for alternative treatments, such as probiotics, has intensified. Objective: This study investigated the anti-inflammatory potential of *Lactiplantibacillus plantarum* 6.2 (Lp62), administered orally in a murine ALI model.

**Materials and Methods:**

Thirty male Swiss mice (N = 30) were randomly assigned to five groups (n = 6): negative control, healthy supplemented with Lp62 (Lp62), ALI, ALI supplemented with Lp62 (ALI+Lp62), and ALI treated with Dexamethasone (Dexa). Lp62 (10⁹ CFU/mL) was administered daily by oral gavage for one week. ALI was induced by intranasal instillation of LPS under anesthesia. Lungs were collected 24 h later for macroscopic examination. Histological evaluation used hematoxylin–eosin staining, and immunohistochemistry included anti-TLR4, anti-NF-κB, and anti-IL-17 antibodies. Total protein in bronchoalveolar lavage fluid (BALF) was measured by the Biuret method, and IL-1β by ELISA. Data were analyzed by one-way ANOVA with Tukey’s post hoc test (p < 0.05).

**Results:**

Lp62 reduced cell migration, protein exudation, and IL-1β release in BALF, as well as macroscopic lung lesions, and attenuated histological inflammatory infiltration, increasing alveolar spaces. It also decreased TLR4, NF-κB, and IL-17 activation.

**Conclusion:**

Lactiplantibacillus plantarum 6.2 is a promising therapeutic approach for ALI by inhibiting the TLR4/NF-κB pathway and reducing neutrophil recruitment via IL-17–mediated signaling in mice.

## Introduction

Acute lung injury (ALI) is defined as a syndrome that affects the alveolar–capillary barrier, impairing gas exchange (Rotta et al. 2015) and leading to substantial morbidity and mortality (Bernardo et al. 2022). Globally, the incidence of ALI and acute respiratory distress syndrome (ARDS) varies widely across intensive care units (ICUs), with estimates ranging from 2% to 26%, depending on population characteristics, diagnostic criteria, and regional healthcare resources (Cavalcanti et al. 2024). These international data highlight the clinical relevance and heterogeneity of ALI across different settings.

In Brazil, epidemiological studies on ALI/ARDS remain limited. One of the few available local reports, conducted in a tertiary hospital ICU, identified pneumonia, shock, and sepsis as the most common risk factors among patients who developed ALI. Although this study does not represent national or global incidence, it provides valuable insight into the Brazilian clinical context and underscores the scarcity of regional epidemiological data (Huang et al. 2023). Diagnostic challenges and limitations in therapeutic management contribute to its classification as a public health concern (Pereira et al. 2025).

Mechanical ventilation is the most used treatment for ALI (Rotta et al. 2015). However, therapeutic strategies capable of significantly reducing mortality remain limited, and no definitive pharmacological therapy has been established. Consequently, the primary goals of ALI management include controlling pulmonary edema and ensuring adequate gas exchange (Bernardo et al. 2022).

Under these circumstances, the search for new therapeutic approaches, such as the use of probiotic lactobacilli, including *Lactiplantibacillus plantarum*, have gained increasing attention for their therapeutic applications in inflammatory diseases such as combined allergic rhinitis and asthma syndrome (Cavalcanti et al. 2024), ulcerative colitis (Huang et al. 2023), osteoporosis (Pereira et al. 2025), osteomyelitis (Carrega et al. 2023), among others (Ren et al. 2015, Ong et al. 2020). These lactic acid bacteria exhibit notable immunomodulatory properties, suggesting potential benefits in attenuating inflammation diseases (Ramos et al. 2022).

The *Lactobacillus plantarum* 6.2 (Lp62) strain, also referred to as TcUESC02 (Nascimento et al. 2026) and originally obtained from cocoa fermentation, exhibits multiple properties that support its use as a probiotic. These include: (1) ability to withstand gastrointestinal transit (Saito et al. 2014); (2) pronounced cell-surface hydrophobicity and strong autoaggregation capacity (Pessoa et al. 2017, Silva et al. 2023); (3) capability to coaggregate with and inhibit pathogens such as *Staphylococcus aureus*, *Escherichia coli*, and *Gardnerella vaginalis* (Ramos et al. 2022, Pessoa et al. 2017, Melo et al. 2016, Oliveira et al. 2022); (4) robust adherence to epithelial cells of the host (Pessoa et al. 2017, Silva et al. 2023); (5) sensitivity to antimicrobials of clinical relevance (Pessoa et al. 2017, Silva et al. 2023); and (6) lack of virulence factors, including hemolysin, gelatinase, and collagenase (Silva et al. 2023).

In addition, Lp62 has demonstrated immunomodulatory activity both in vitro and in vivo. This strain reduces the production of proinflammatory cytokines, including IL-8, IL-1β, IL-17, and TNF, while enhancing the secretion of anti-inflammatory mediators such as IL-10 (Santos et al. 2016). Moreover, maternal supplementation with Lp62 during late gestation and lactation has been shown to improve milk nutritional composition, potentially contributing to increased pup body weight. Elevated levels of TGF-β in the milk are thought to confer additional immunological benefits to the offspring (Messias et al. 2019). Oral administration of Lp62 showed therapeutic potential in experimental medication-related osteonecrosis of the jaw (MRONJ) by enhancing bone and epithelial repair, reducing NF-κB–mediated inflammatory signaling, and increasing TGF-β–associated regenerative activity (Nascimento et al. 2026). Given these biological properties, it is hypothesized that Lp62 may offer therapeutic advantages in the treatment of ALI.

This study proposes that oral supplementation with Lp62, a strain isolated from cocoa fermentation, exerts anti-inflammatory effects in an LPS-induced murine model of ALI, which reproduces key features of the disease’s pathophysiology in humans.

Therefore, this study aimed to evaluate the anti-inflammatory potential of the Lp62 strain, administered orally in a murine model of LPS-induced murine model of ALI.

## Materials and methods

### Ethical considerations

This study was approved by the Institutional Ethics Committee for Animal Research of the Federal University of Paraíba (CEUA/UFPB; protocol no. 3188131223; ID 002108) and conducted in accordance with the principles of Law 11.794 (October 8, 2008), Decree 6.899 (July 15, 2009), and the guidelines issued by the National Council for Animal Experimentation Control (CONCEA) (CONCEA ).

### Animals

Thirty male Swiss mice (*Mus musculus*) (*N* = 30) 30 days old and weighing approximately 30 g, were obtained from the Animal Production Unit of the Institute of Pharmaceutical and Drug Research (IPeFarM), Federal University of Paraíba (UFPB). Animals were maintained under controlled conditions (23 ± 2 °C; 50% relative humidity) on a 12 h light/dark cycle, with *ad libitum* access to filtered water and a standard pellet diet (Nuvilab^®^, Quimtia S/A, Paraná, Brazil).

The mice were randomly assigned to five groups (*n* = 6 per group): negative control (healthy), healthy mice supplemented with Lp62 (Lp62), experimental acute lung injury (ALI), ALI supplemented with Lp62 (ALI+Lp62), and experimental ALI treated with 2 mg/kg of Dexamethasone (Dexa) —Ache^®^, São Paulo, SP, Brazil.

### Induction of ALI

The mice were first anesthetized with ketamine (80 mg/kg) and xylazine (10 mg/kg) and then received a single dose from 10 µg/kg of LPS (*Escherichia coli*, Sigma-Aldrich^®^) diluted in 60 µL of phosphate-buffered saline (PBS, pH 7.2), administered via nasal instillation (Pacheco de Oliveira et al. 2015).

### Microorganism

The *Lactiplantibacillus plantarum* 6.2 (Lp62) strain, originally isolated from spontaneous cocoa fermentation in the Ilhéus and Itabuna regions of Bahia, Brazil (Messias et al. 2019), was obtained from the State University of Santa Cruz (UESC).

The strain was stored at − 80 °C in De Man, Rogosa, and Sharpe (MRS) broth supplemented with 15% glycerol and reactivated in MRS broth at 37 °C for 16 h under aerobic conditions. Molecular identification of *L. plantarum* was confirmed using 16 S rDNA sequencing data kindly provided by Masterfoods from previous experiments (Selis et al. 2021). Identification was performed by amplification of the 16 S rDNA gene using primers 27f and 1512R, followed by sequencing with the 27f primer.

For RAPD-PCR, the isolate was cultured in MRS broth for 20 h at 37 °C, and total genomic DNA was extracted using the GenElute^®^ Bacterial Genomic DNA Kit (Sigma–Aldrich, USA) according to the manufacturer’s instructions. DNA concentration was determined using a NanoDrop spectrophotometer and adjusted to 20 ng/µL. Amplification was performed with primers OPL01 (GGCATGACCT), OPL02 (TGGGCGTCAA), OPL04 (GACTGCACAC), OPL05 (ACGCAGGCAC), OPL14 (GTGACAGGCT), and OPL20 (TGGTGGACCA), following Todorov et al. (2010) (Todorov et al. 2010). A 1 kb DNA ladder (Thermo Fisher Scientific, Fermentas) was used as a molecular marker.

*Oral supplementation with* Lactiplantibacillus plantarum *6.2*.

The Lp62 strain was cultured in MRS broth for 24 h at 35 ± 2 °C and plated on MRS agar. Colonies with typical morphology were isolated and confirmed by Gram staining.

For oral supplementation, Lp62 cells were grown in MRS broth for 24 h at 35 ± 2 °C in 15 mL tubes. Cultures were centrifuged at 2,000 rpm for 10 min at room temperature (Centrifuge 80-2dB 15 mL, Centrilab, Pindamonhangaba, São Paulo, Brazil). The resulting pellet was resuspended in PBS to yield a concentration of 10⁹ CFU/mL. Absorbance was measured at 560 nm using a spectrophotometer (Spectro 100, Thermo Fisher Scientific, Waltham, MA, USA), and cell counts were confirmed by serial dilution and plating on solid MRS agar (Santos et al. 2016, Messias et al. 2019).

The Lp62 suspension (10⁹ CFU/mL) was administered by oral gavage (300 µL/day) for seven consecutive days before induction of ALI (Nascimento et al. 2026, Santos et al. 2016).

**Fig. 1:**
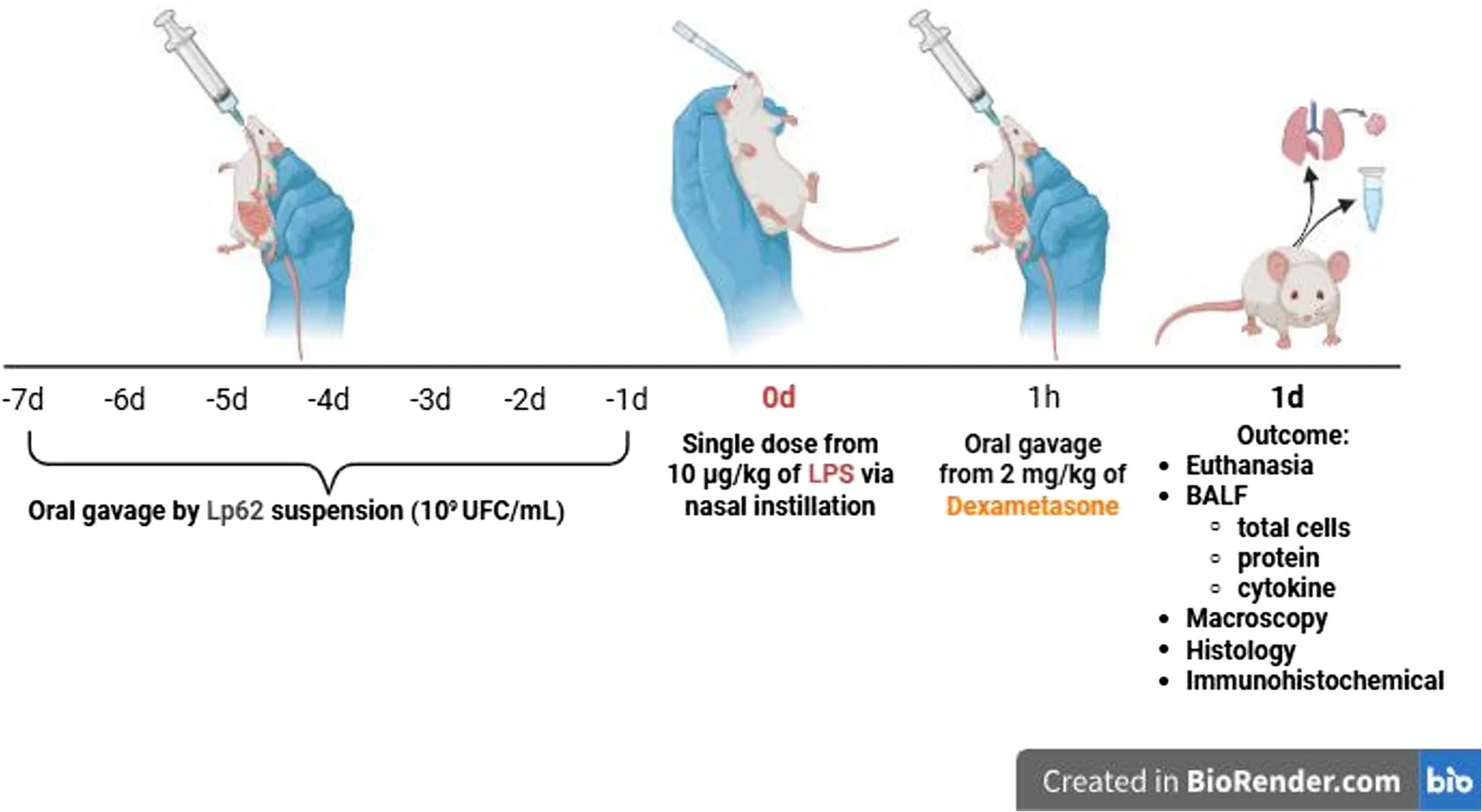
Experimental timeline of the murine model of acute lung injury (ALI) and therapeutic interventions. Mice received daily oral supplementation with Lactiplantibacillus plantarum 6.2 (Lp62; 10⁹ CFU/mL; 300 µL) for seven consecutive days (days − 7 to − 1). On day 0, under anesthesia, animals were challenged with intranasal instillation of LPS (10 µg/kg) to induce ALI. One hour after LPS administration, mice in the pharmacological control group received dexamethasone (2 mg/kg, orally). At 24 h post LPS challenge (day + 1), animals were euthanized, followed by bronchoalveolar lavage fluid (BALF) collection for quantification of total cells, protein concentration, and IL 1β levels. Subsequently, lungs were harvested for macroscopic evaluation, histological analysis (H&E), and immunohistochemistry for TLR4, NF κB, and IL 17. Created with BioRender.com (free version)

The complete experimental timeline, including the schedule of Lp62 supplementation, LPS challenge, dexamethasone administration, BALF collection, euthanasia, and tissue harvesting, is presented in Fig. 1.

### Euthanasia

At the end of the ALI induction and Lp62 suplementation, animals were euthanized according to the Guidelines for Euthanasia Practices of the National Council for Animal Experimentation Control (CONCEA). A combination of ketamine (180 mg/kg) and xylazine (30 mg/kg) (Syntec, São Paulo, Brazil) was administered intraperitoneally (Flecknell 2009), and death was confirmed by the absence of corneal reflex (CONCEA ).

### Inflammatory cells in bronchoalveolar lavage fluid (BALF)

BALF were collected 24 h after the LPS challenge. An intratracheal cannula was administered to the lung with 1.5 mL of PBS, the BALF was harvested and the pellet was used for leukocyte counting in a Neubauer chamber, using the Turk solution under an optical microscope (40×—BX40, OLYMPUS, Tokyo, Japan).

### Macroscopic examination of lungs

After obtaining the BALF, the lungs were surgically removed, examined under appropriate lighting and tissues were dissected, photographed, and evaluated for anatomopathological alterations, such as color, appearance, texture, weight, and size of the lesions.

### Protein concentration in the BALF

Total protein in BALF concentration was determined by a colorimetric reaction using the Biuret method. For this analysis, the Labtest Diagnóstica commercial kit (Lagoa Santa, Minas Gerais, Brazil) was employed in conjunction with the CHEMWELL‑T automated biochemical analyzer.

### Cytokine quantification in the BALF

The pro-inflammatory cytokine IL-1β was quantified in BALF using ELISA kit, according to the manufacturer’s instructions (Invitrogen Thermo Fisher Scientific, Viena, Austria). Absorbance was measured at 450 nm using an Epoch™ microplate spectrophotometer (Agilent BioTek Instruments, USA), and cytokine concentrationwas calculated based on the standard curve for each analyte.

### Histological processing

Lungs were fixed in 10% neutral-buffered formalin at room temperature (23 ± 2 °C) for 48 h and lesions were sectioned, dehydrated in graded ethanol (70%, 80%, 90%, and 100%), cleared in xylene, and embedded in paraffin (maximum 60 °C). Section  (4 μm thick) were mounted on glass slides, deparaffinized, and rehydrated in descending alcohol concentrations. Sections were stained with hematoxylin and eosin (H&E) (Lima et al. 2023).

### immunohistochemical analysis

Formalin-fixed, paraffin-embedded tissues were sectioned at 4 μm. Antigen retrieval was performed in citrate buffer (pH 6.0; EasyPath Diagnostics) using a Pascal pressurized chamber (Dako Cytomation, Denmark). Endogenous peroxidase activity was blocked with 3% hydrogen peroxide for 30 min at room temperature (EasyPath Diagnostics). After protein blocking, sections were incubated overnight in a humid chamber with primary antibodies against TLR4 (Cloud-Clone Corp., PAA753, polyclonal antibody; 1:100), NF-κB (Cloud-Clone Corp., PAB82RA01, polyclonal antibody; 1:100), and IL-17 (Booster, A00421-2, polyclonal antibody; 1:100).

Subsequently, biotinylated secondary antibodies (goat anti-rabbit and anti-mouse; 1:100; Link System-HRP, Dako 4061) were applied. Antigen detection was visualized with 0.024% 3,3′-diaminobenzidine (DAB-HRP; EasyPath Diagnostics), followed by counterstaining with Harris hematoxylin (Pontes et al. 2024, Atomoxetine attenuates methotrexate-induced lung injury in rats implicating TLR4/NF-κB ).

### Histomorphometric analysis

H&E stain and immunostained sections were examined under a light microscope (Eclipse Ci-L, Nikon, Tokyo, Japan) at 40× magnification. Images were captured using a DS-Ri2 camera and analyzed with NIS Elements D software (version 4.00, Nikon).

Binary images were generated, and inflammatory areas were quantified in µm² and expressed as mean ± standard error of the mean (SEM). TLR4, NF-κB, and IL-17-positive areas were quantified using Image-Pro Plus software (Media Cybernetics, Rockville, MD, USA). Pixels corresponding to brown tones (immunostaining) were selected for analysis, and results were expressed as mean ± SEM (Nascimento et al. 2026, Pontes et al. 2024).

### Statistical analysis

All data are presented as mean ± standard error of the mean (SEM). Prior to hypothesis testing, the distribution of each dataset was assessed using the Shapiro–Wilk normality test. Because all variables met the assumptions of normality, parametric analyses were applied.

Sample size (*n* = 6 animals per group) was determined a priori using the sample size calculator provided by the University of São Paulo (USP), which is specifically designed for studies involving multiple independent groups. The calculation was based on expected effect sizes derived from previous studies using *Lactiplantibacillus plantarum* 6.2 and similar inflammatory endpoints, ensuring a statistical power of at least 80% to detect biologically relevant differences.

For each predefined outcome (BALF cellularity, protein concentration, IL-1β levels, histomorphometric measurements, and immunohistochemical quantification), comparisons among experimental groups were performed using one-way analysis of variance (ANOVA), followed by Tukey’s post hoc test. Tukey’s procedure provides built-in correction for multiple pairwise comparisons within each endpoint, thereby controlling type I error at the familywise level for each outcome domain. Because each endpoint represents a distinct and biologically justified measure of lung injury severity, no global correction across unrelated outcome families was applied.

All statistical analyses were performed using GraphPad Prism^®^ version 8.0.2 (GraphPad Software, San Diego, CA, USA), and significance was set at *p* < 0.05.

## Results

### Lp62 ameliorates the leukocyte infiltration in LPS-Induced ALI

LPS administration induced intense leukocyte migration into the lungs. The ALI+Lp62 group showed an approximately 50% reduction in cell migration to the BALF compared with the ALI group, like what was observed for the dexa group. In all other experimental conditions, cell migration to the bronchoalveolar lavage remained comparable to that of the healthy group, with no notable differences.

**Fig. 2:**
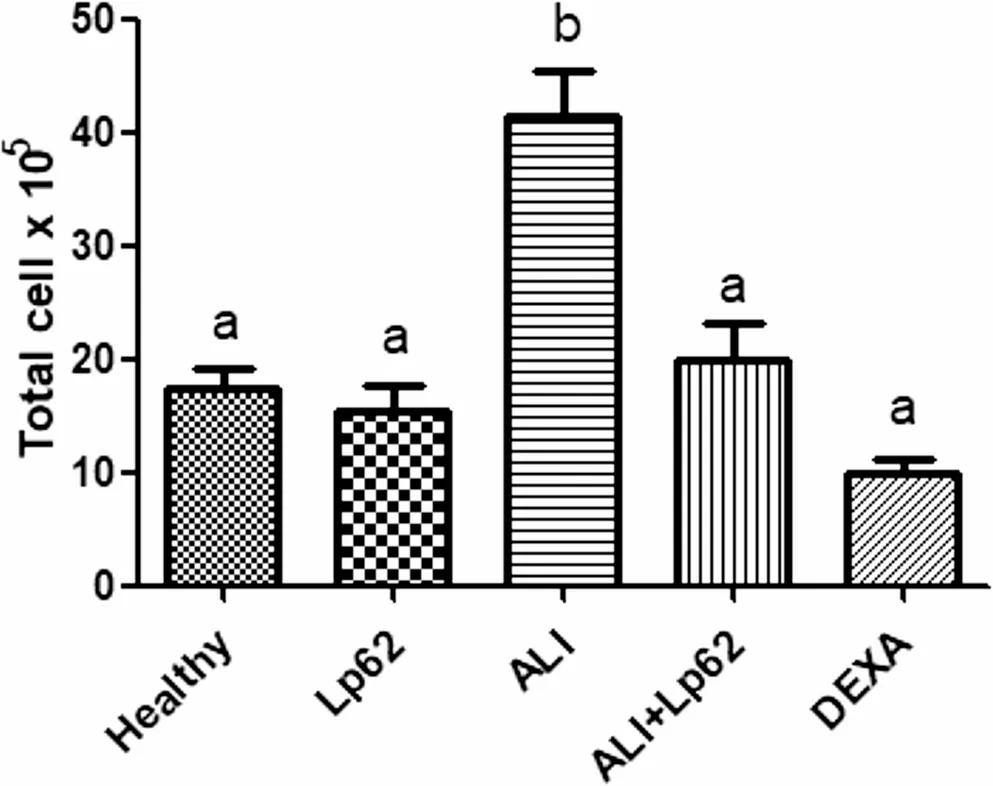
Representative total cell number in the bronchoalveolar lavage fluid (BALF) was quantified in the Neubauer camera. Healthy: negative control, Lp62: healthy mice supplemented with Lp62, ALI: experimental acute lung injury, ALI+Lp62: ALI supplemented with Lp62 and Dexa: acute lung injury treated with Dexamethasone. Different letters denote significant differences (*p* < 0.05; one-way ANOVA, Tukey’s post-test)

### Protein concentration in the BALF

**Fig. 3:**
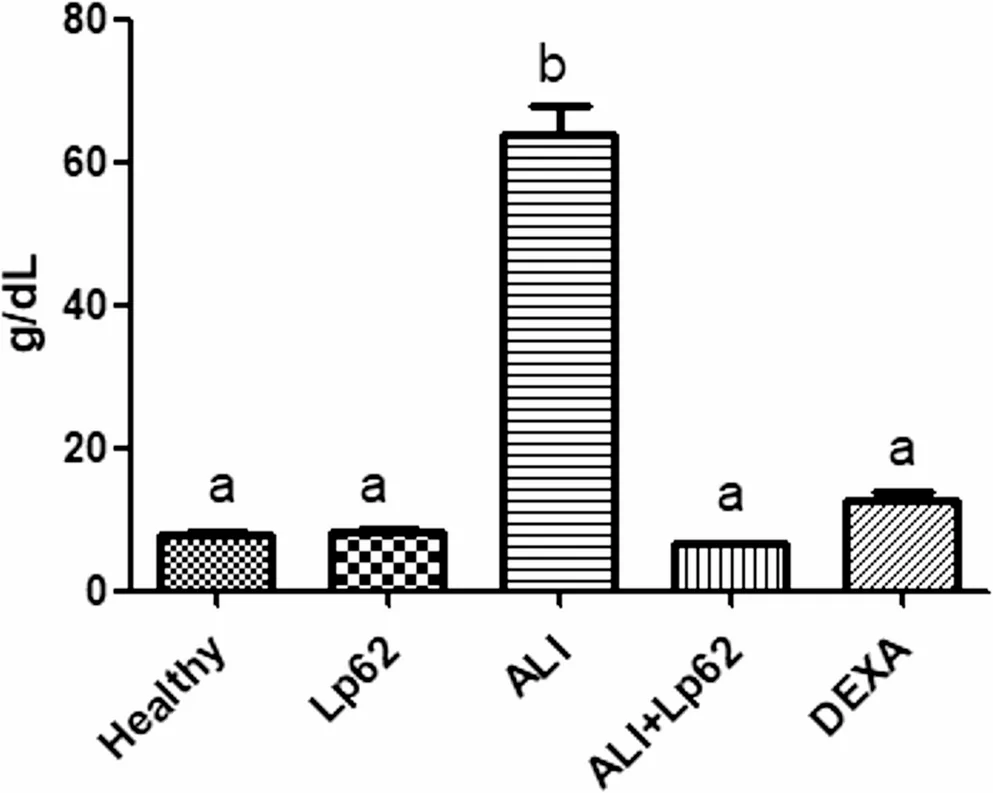
Representative protein concentration in the bronchoalveolar lavage fluid (BALF). Healthy: negative control, Lp62: healthy mice supplemented with Lp62, ALI: experimental acute lung injury, ALI+Lp62: ALI supplemented with Lp62 and Dexa: acute lung injury treated with Dexamethasone. Different letters denote significant differences (*p* < 0.05; one-way ANOVA, Tukey’s post-test)

After evaluating the effect of treating mice with Lp62 on cellular infiltrate in the ALI model, the next step was to assess protein and IL-1β levels in BALF. Corroborating the data obtained in Fig. 3, the protein and IL-1β dosage data in BALF show a significant increase in protein exudate and IL-1β level in the ALI group, while treatment with Lp62 reduced exudate and IL-1β to levels comparable to control animals and like what was observed for dexamethasone.

### Macroscopic evaluation of lungs

**Fig. 4:**
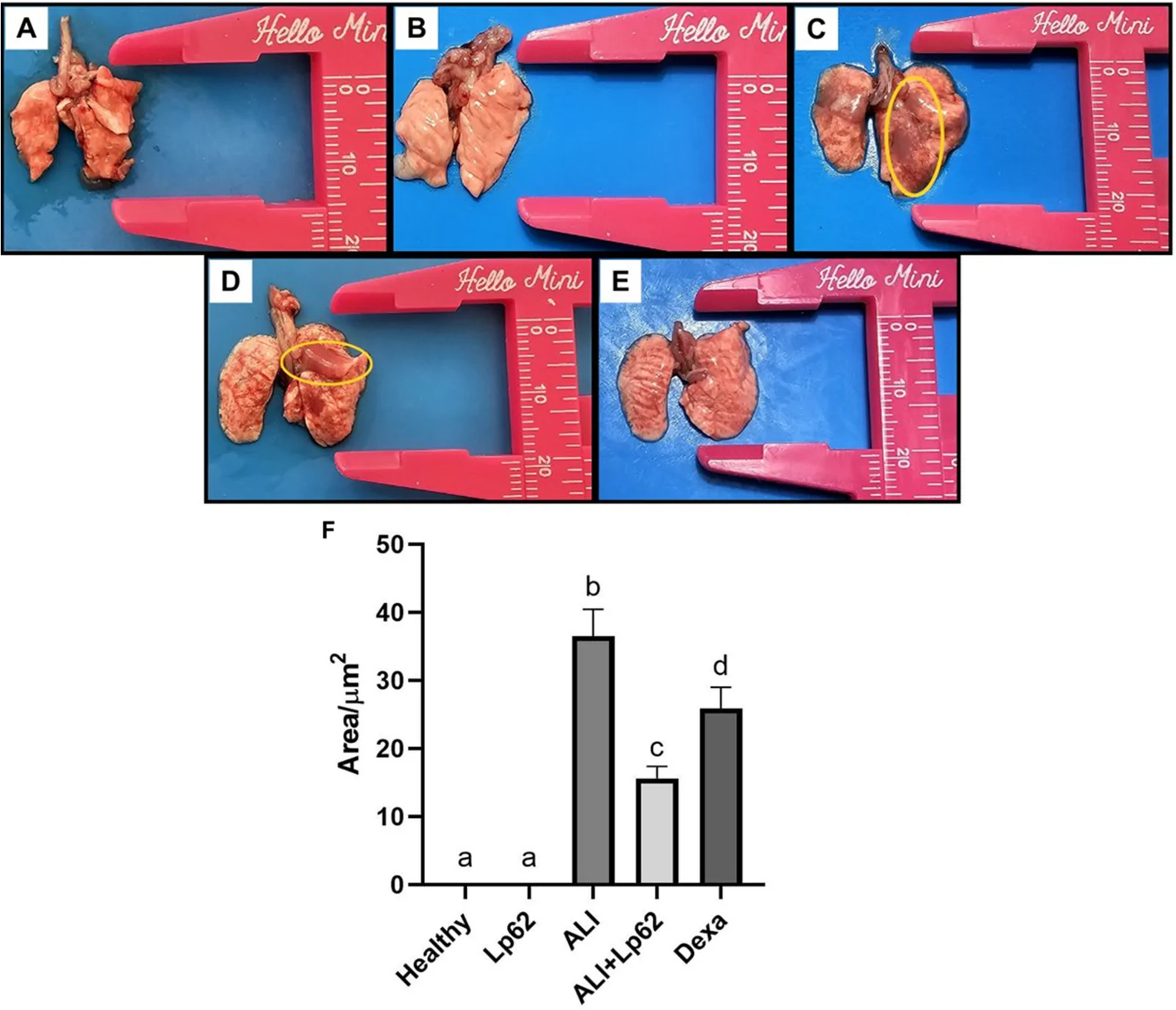
Representative macroscopic appearance of lungs from each experimental group. Healthy (**A**): negative control, Lp62 (**B**): healthy mice supplemented with Lp62, ALI (**C**): experimental acute lung injury, ALI+Lp62 (**D**): ALI supplemented with Lp62 and Dexa (**E**): acute lung injury treated with Dexamethasone. Yellow circle: macroscopic areas of acute lung injury. Graph (**F**): Histomorphometric analysis of acute lung injury area across experimental conditions. Data are expressed as mean ± SEM (*n* = 6 measurements/group). Different letters denote significant differences (*p* < 0.05; one-way ANOVA, Tukey’s post-test)

The Healthy and Lp62 groups exhibited lungs with a whitish-tan coloration and a smooth, homogeneous surface, consistent with normal pulmonary morphology (Fig. 4a and b). In the ALI group, macroscopic areas of acute injury, represented by wine-colored, edematous, and irregular areas, were observed (Fig. 4c, yellow circle). The ALI+Lp62 group showed a marked reduction in lesions compared with the ALI group, with lighter coloration, a more homogeneous appearance, and a smooth surface (Fig. 4d). The DEXA group also demonstrated a decrease in injured areas relative to the ALI group, with clearer and less irregular regions (Fig. 4e).

Morphometric analysis of the wine-colored pulmonary areas shows that the ALI+Lp62 group exhibited an approximately 57% reduction in lesion size compared with the ALI group (Fig. 4f).

### Histological and histomorphometric evaluation of lung

**Fig. 5:**
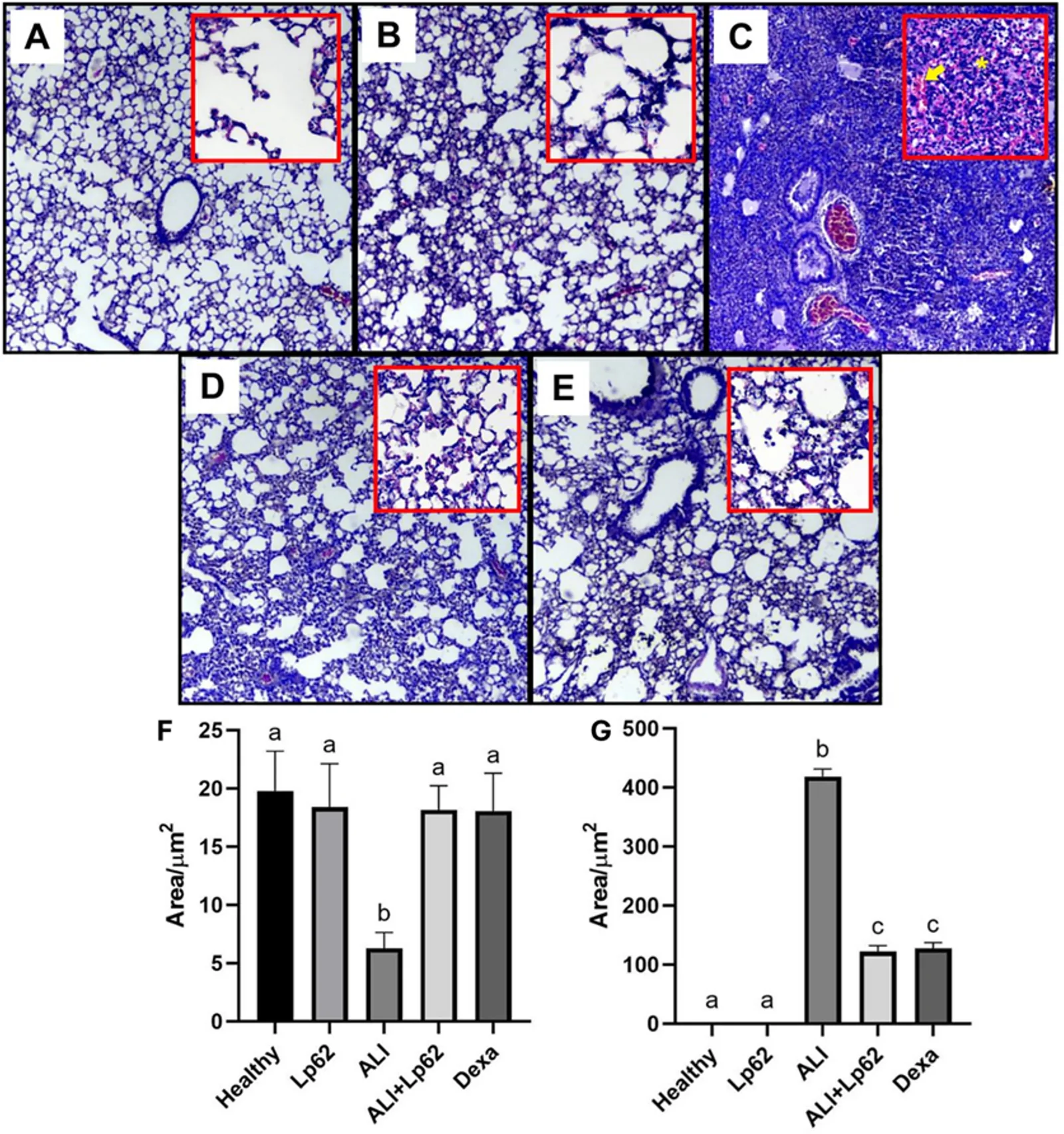
Representative histological sections of lung stained with hematoxylin and eosin (H&E) showing alveolar opening and neutrophil migration. Healthy (**A**): negative control, Lp62 (**B**): healthy mice supplemented with Lp62, ALI (**C**): experimental acute lung injury, ALI+Lp62 (**D**): ALI supplemented with Lp62 and Dexa (**E**): acute lung injury treated with Dexamethasone. Yellow arrow: hyperemia. Histomorphometric analysis of alveolar opening lung area across experimental conditions (**F**). Histomorphometric analysis of neutrophil migration area across experimental conditions (**G**). Data are expressed as mean ± SEM (*n* = 6 measurements/group). In F and G: different letters denote significant differences (*p* < 0.05; one-way ANOVA, Tukey’s post-test)

The Healthy and Lp62 groups exhibited preserved epithelium, with adequate alveolar opening and air spaces consistent with normal pulmonary morphology (Fig. 5a and b). In contrast, the ALI group showed hyperemia, intense inflammatory infiltrate, and reduced alveolar opening (Fig. 5c). In the ALI+Lp62 and DEXA groups, larger alveolar spaces and fewer inflammatory cells were observed compared with the ALI group (Fig. 5d and e).

Morphometric analysis of the alveolar opening area, presented in Fig. 5f, shows that the ALI+Lp62 group exhibited an increase of approximately 65% relative to the ALI group. Similarly, morphometric analysis of neutrophil migration area, shown in Fig. 2g indicates that the ALI+Lp62 group displayed a reduction in cellular migration of about 71% compared with the ALI group.

### Immunohistochemical evaluation for Toll-like 4

**Fig. 6:**
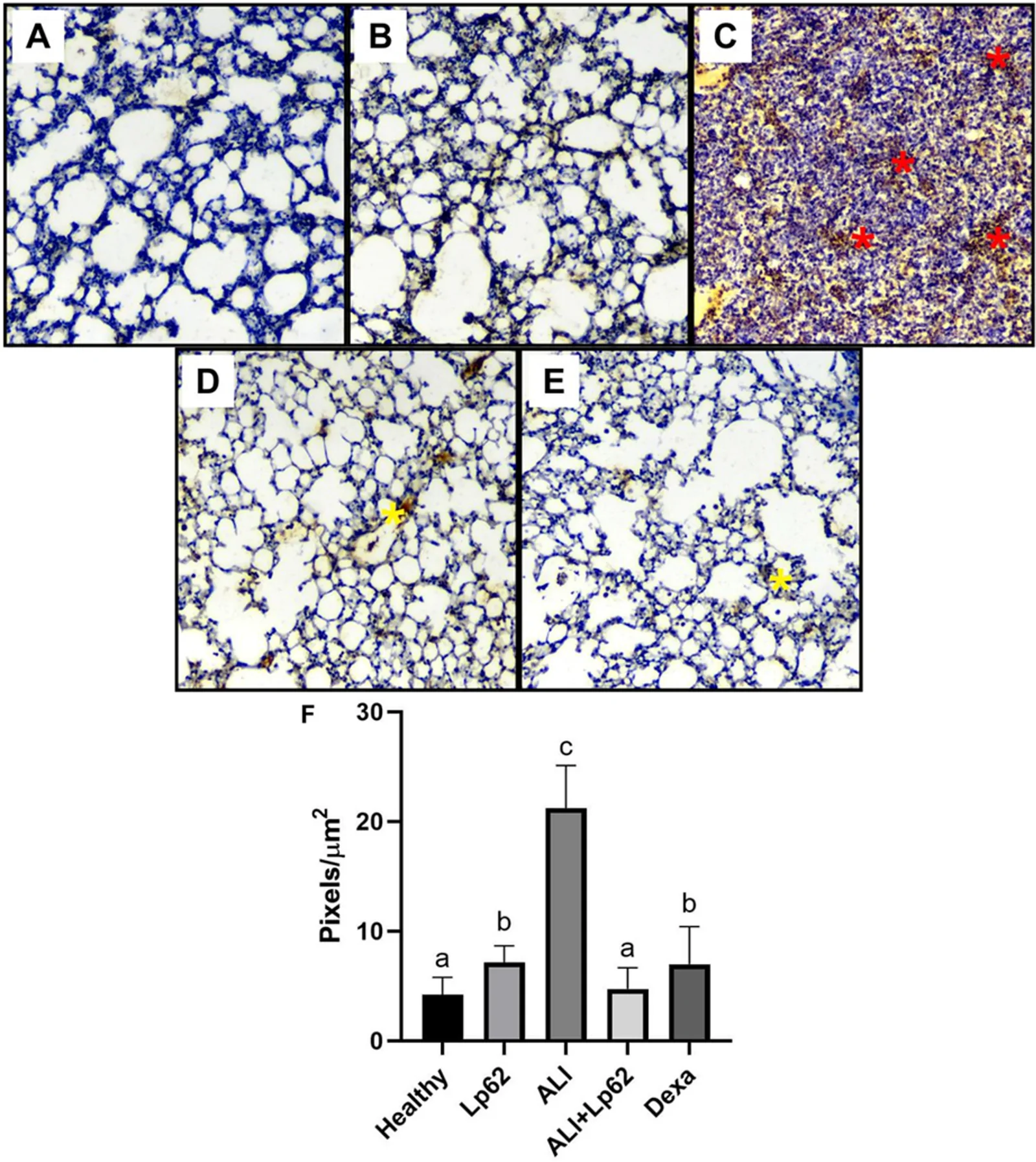
Representative immunohistochemical staining for Toll-Like 4 counterstained with hematoxylin. Groups: Healthy (**A**): negative control, Lp62 (**B**): healthy mice supplemented with Lp62, ALI (**C**): experimental acute lung injury, ALI+Lp62 (**D**): ALI supplemented with Lp62 and ALI+Dexa (**E**): acute lung injury treated with Dexamethasone. Red asterisks: intense and positive Toll-Like 4 staining in lung tissue. Yellow asterisks: low-intense and positive Toll-Like 4 staining in lung tissue Magnification: 20×. Quantitative analysis of toll-like 4-positive areas under different experimental conditions (**F**). Data are presented as mean ± SEM. Different letters denote significant differences (*p* < 0.05; one-way ANOVA, Tukey’s post-test)

All experimental groups exhibited cell areas of lung tissue showing brown immunostaining indicative of *Toll-like 4* presences. The Healthy and Lp62 groups showed mild staining (Fig. 6a and b). The ALI group, in contrast, exhibited intense and diffuse staining, particularly within the inflammatory infiltrate (Fig. 6c). The ALI+Lp62 and ALI+DEXA groups displayed staining in multiple regions of the pulmonary parenchyma and within the inflammatory infiltrate, but with lower intensity compared with the ALI group (Fig. 6d and e).

Histomorphometric quantification revealed that Toll-like 4 immunoreactivity was significantly lower in the Healthy, Lp62, ALI+Lp62 and ALI+DEXA group compared to the ALI group. The ALI+Lp62 group reduced staining positivity by 72.31% compared with the ALI group (Fig. 6f).

### *Immunohistochemical evaluation for* NF-κB

**Fig. 7:**
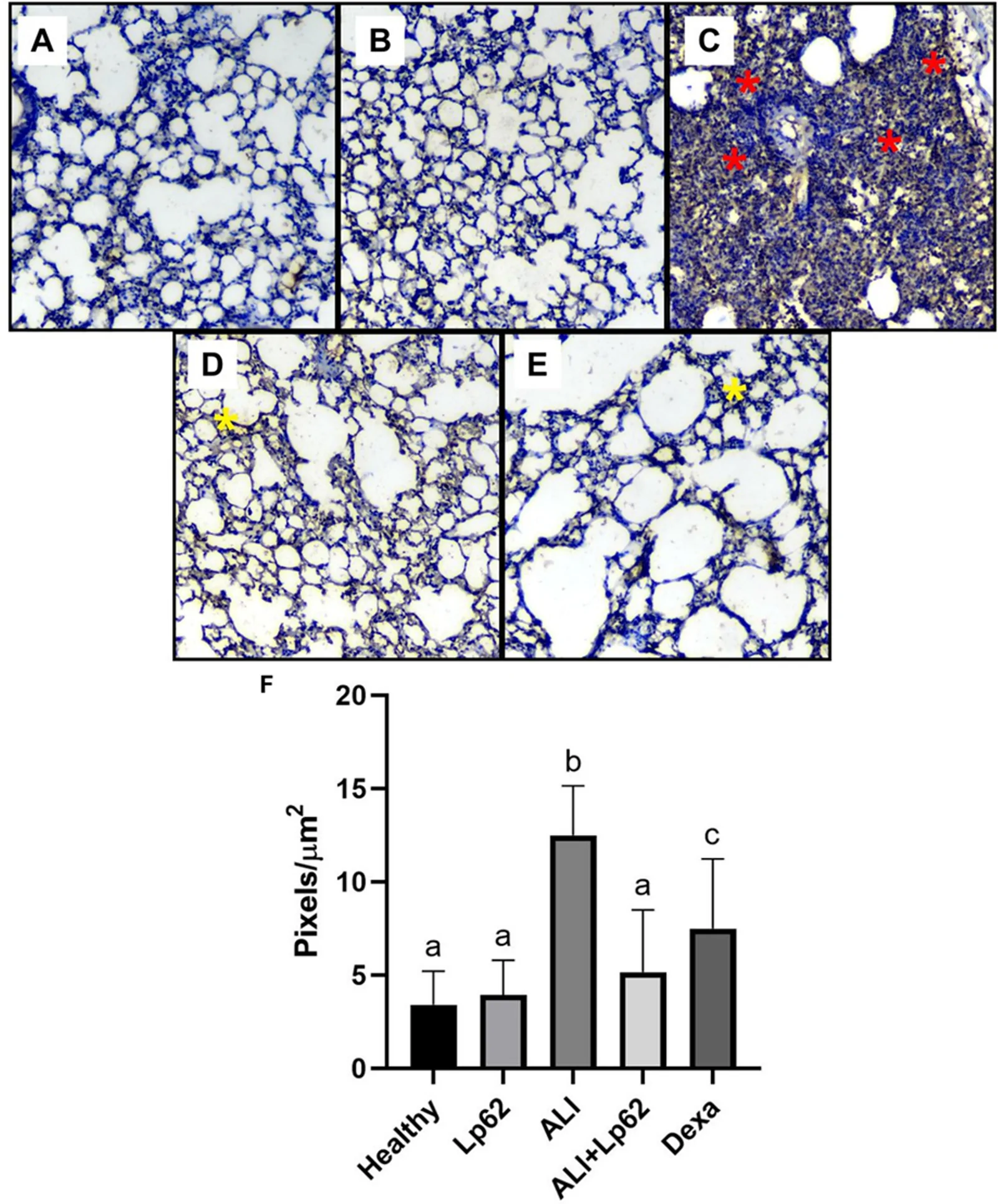
Representative immunohistochemical staining for NF-κB counterstained with hematoxylin. Groups: Healthy (**A**): negative control, Lp62 (**B**): healthy mice supplemented with Lp62, ALI (**C**): experimental acute lung injury, ALI+Lp62 (**D**): ALI supplemented with Lp62 and Dexa (**E**): acute lung injury treated with Dexamethasone. Red asterisks: intense and positive NF-κB staining in lung tissue. Yellow asterisks: low-intense and positive NF-κB staining in lung tissue Magnification: 20×. Quantitative analysis of NF-κB-positive areas under different experimental conditions (**F**). Data are presented as mean ± SEM. Different letters denote significant differences (*p* < 0.05; one-way ANOVA, Tukey’s post-test)

All experimental groups exhibited cell areas of lung tissue showing brown immunostaining indicative of NF-κB presences. The Healthy and Lp62 groups showed mild staining (Fig. 7a and b). The ALI group, in contrast, exhibited intense and diffuse staining, particularly within the inflammatory infiltrate (Fig. 7c). The ALI+Lp62 and DEXA groups displayed staining in multiple regions of the pulmonary parenchyma and within the inflammatory infiltrate, but with lower intensity compared with the ALI group (Fig. 7d and e).

Histomorphometric quantification revealed that NF-κB immunoreactivity was significantly lower in the Healthy, Lp62, ALI+Lp62 and DEXA group compared to the ALI group. The ALI+Lp62 group reduced staining positivity by 70.11% compared with the ALI group (Fig. 7f).

### *Immunohistochemical evaluation for* IL-17

**Fig. 8:**
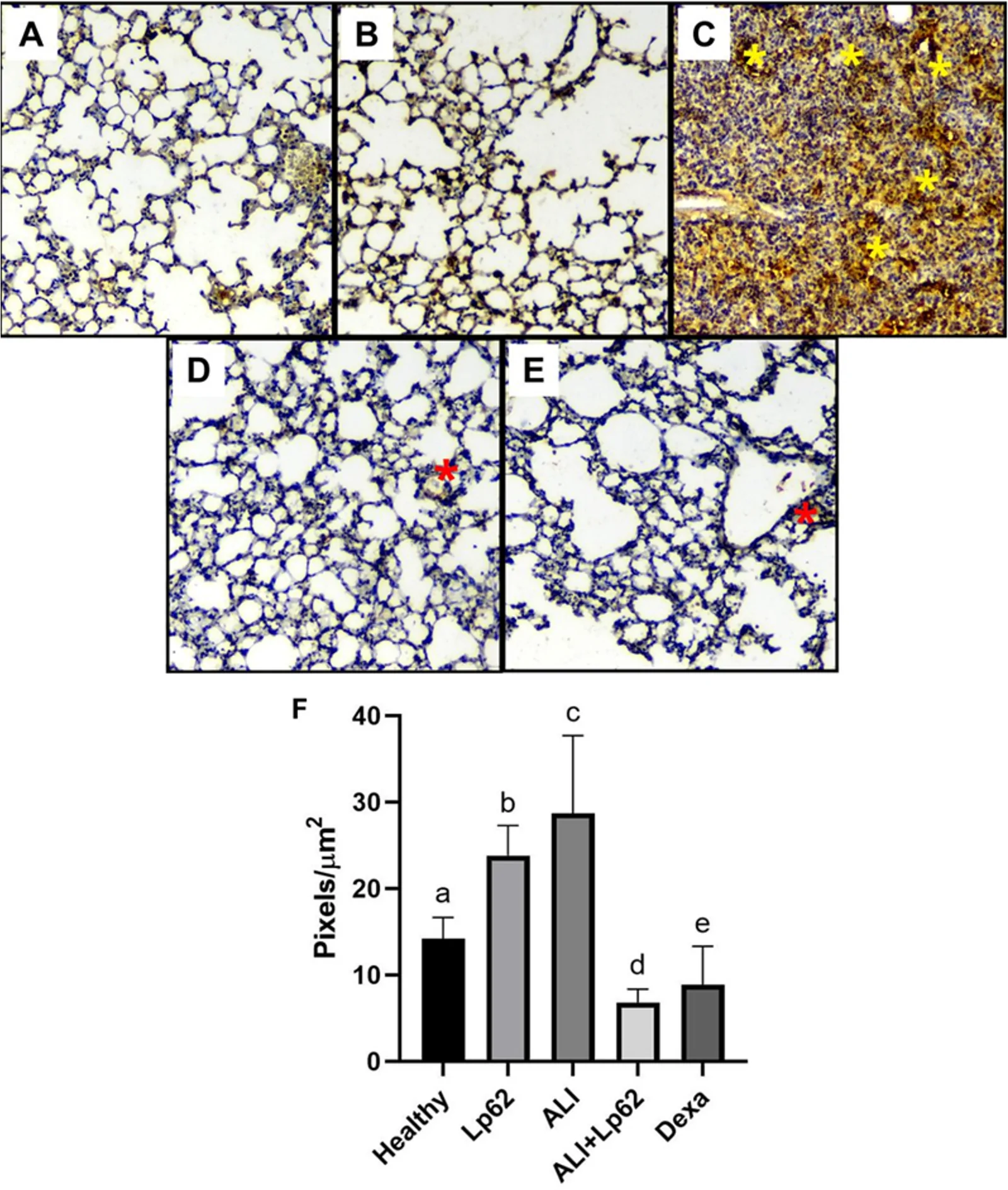
Representative immunohistochemical staining for IL-17 counterstained with hematoxylin. Groups: Healthy (**A**): negative control, Lp62 (**B**): healthy mice supplemented with Lp62, ALI (**C**): experimental acute lung injury, ALI+Lp62 (**D**): ALI supplemented with Lp62 and ALI+Dexa (**E**): acute lung injury treated with Dexamethasone. Red asterisks: intense and positive IL-17 staining in lung tissue. Yellow asterisks: low-intense and positive IL-17 staining in lung tissue. Magnification: 20×. Quantitative analysis of IL-17-positive areas under different experimental conditions (**F**). Data are presented as mean ± SEM. Different letters denote significant differences (*p* < 0.05; one-way ANOVA, Tukey’s post-test)

All experimental groups exhibited cell areas of lung tissue showing brown immunostaining indicative of IL-17 presences. The Healthy and Lp62 groups showed mild staining (Fig. 8a and b). The ALI group, in contrast, exhibited intense and diffuse staining, particularly within the inflammatory infiltrate (Fig. 8c). The ALI+Lp62 and ALI+DEXA groups displayed staining in multiple regions of the pulmonary parenchyma and within the inflammatory infiltrate, but with lower intensity compared with the ALI group (Fig. 8d and e).

Histomorphometric quantification revealed that IL-17 immunoreactivity was significantly lower in Healthy, Lp62, ALI+Lp62 and ALI+DEXA group compared to the ALI group. The ALI+Lp62 group reduced staining positivity by 76.28% compared with the ALI group (Fig. 8f).

### Levels of 1 L-1β in the BALF

**Fig. 9:**
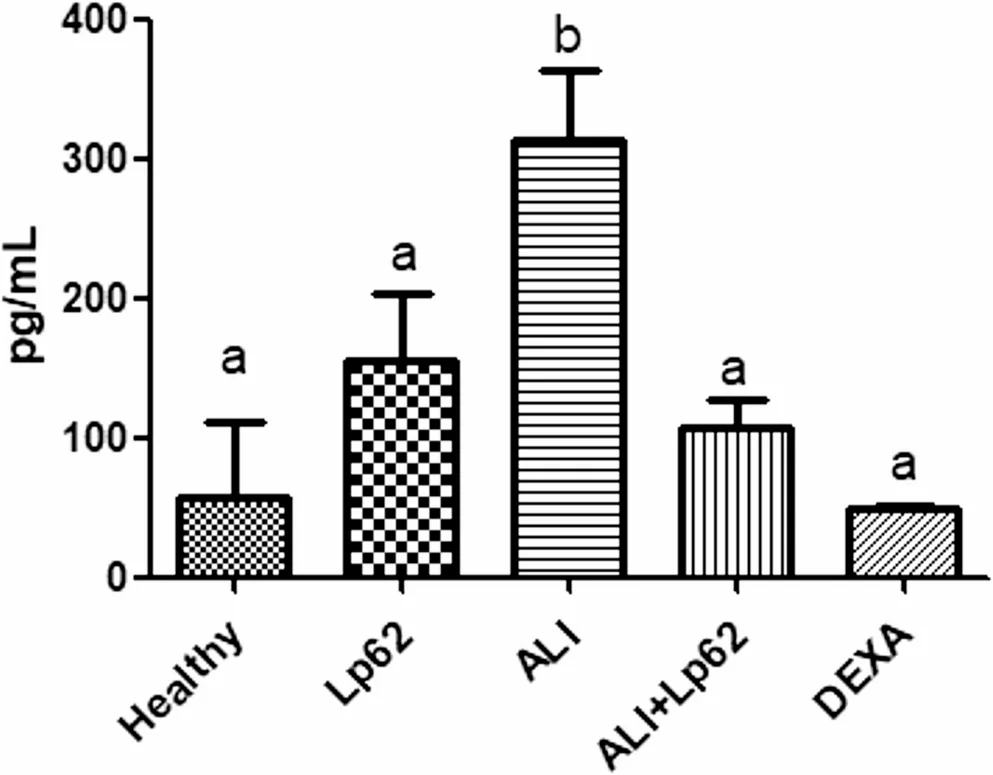
Representative levels of IL-1β in the bronchoalveolar lavage fluid (BALF) for ELISA method. Groups: Healthy: negative control, Lp62: healthy mice supplemented with Lp62, ALI: experimental acute lung injury, ALI+Lp62: ALI supplemented with Lp62 and ALI+Dexa: acute lung injury treated with Dexamethasone. Data are presented as mean ± SEM. Different letters denote significant differences (*p* < 0.05; one-way ANOVA, Tukey’s post-test)

LPS administration induced intense levels of 1 L-1β in the *BALF*. The ALI+Lp62 group exhibited an approximately 33.3% reduction in BALF IL‑1β levels compared with the ALI group. In all other experimental conditions, IL‑1β concentrations in the bronchoalveolar lavage fluid remained comparable to those of the healthy group, with no notable differences (Fig. 9).

## Discussion

Acute lung injury (ALI) remains a significant global clinical challenge, marked by substantial morbidity and mortality. Although considerable progress has been made in elucidating the pathogenesis of ALI over the past decade, current therapeutic approaches are largely limited to supportive measures, including nutritional supplementation, mechanical ventilation, and targeted interventions. This scenario underscores an urgent need to investigate alternative and more effective therapeutic strategies, like *Lactiplantibacillus plantarum* 6.2 (Lp62) (Fan et al. 2018).

This study evaluated the therapeutic effectiveness of oral supplementation with Lp62 in the management of ALI using a murine model. To the best of our knowledge, this is the first study to establish an association between Lp62 and ALI. The experimental model proposed by Oliveira, et al. (2015) (Pacheco de Oliveira et al. 2015) was adapted for this purpose, inducing ALI lesions in Swiss mice (*Mus musculus*) exposed to 10 µg/Kg of LPS for 24 h. The supplementation dosage was determined according to the model proposed by Santos et al. (2016) and Nascimento et al. (2026) (Nascimento et al. 2026, Santos et al. 2016), using a concentration of 10⁹ CFU/mL to ensure Lp62 viability and efficacy.

The findings of this study provide strong evidence for the potential of oral Lp62 supplementation in improving ALI lesions. The LPS‑induced ALI model is marked by intense cellular infiltration during the early hours of injury, with these recruited cells driving the inflammatory response and acting as key effector cells in acute lung injury (Fan et al. 2018). Oral supplementation with Lp62 reduced the total number of cells recovered in the bronchoalveolar lavage fluid (BALF), indicating a clear local modulatory effect. Alveolar edema is a central pathological feature of ALI and is strongly associated with mortality. Increased vascular permeability promotes leukocyte migration and protein leakage into the alveolar space, further impairing gas exchange. Assessment of protein levels in the BALF revealed significant anti‑edematogenic activity of Lp62, supporting its protective role in maintaining alveolar integrity (Antunes et al. 2022).

Macroscopic evaluation of the lungs demonstrated enhanced in the ALI+Lp62 group, characterized by reduction in lesion size compared to the ALI group.

The macroscopic improvement observed in the ALI+Lp62 group aligns with previous studies demonstrating that probiotic supplementation can attenuate pulmonary injury in experimental ALI models. For instance, *Limosilactobacillus reuteri* has been shown to reduce gross lung consolidation and edema in LPS-induced ALI, an effect attributed to its ability to modulate systemic inflammation and preserve alveolar structure (Shen et al. 2023). Similarly, *Lacticaseibacillus rhamnosus* (formely *Lactobacillus rhamnosus*) have been reported to diminish visible lung hemorrhage and tissue darkening, reflecting reduced inflammatory infiltration and vascular leakage on cigarette smoke (CS)-induced COPD murine model (Carvalho et al. 2020). In another study, *Bifidobacterium longum* administration resulted in reduced lung inflammation, inflammatory cytokine expression and adhesion factor expression (Budden et al. 2022). These findings collectively support the concept that probiotics can exert protective effects on lung morphology through gut–lung axis modulation. In this context, the reduced lesion size observed by macroscopic evaluation of the lungs of mice treated with oral Lp62 supplementation, in our study, reinforces the growing evidence that specific commensal microorganisms can attenuate the macroscopic hallmarks of ALI progression, thereby offering a promising adjunctive therapeutic avenue.

Histological confirmation of ALI was achieved through H&E staining, enabling evaluation of assessment of alveolar opening and integrity, as well as the intensity of the inflammatory infiltrate within the pulmonary parenchyma. Promisingly, the ALI+Lp62 group exhibited restoration of pulmonary architecture, as evidenced by reduced neutrophilic infiltration, absence of hyperemia, and consequently greater alveolar opening compared with the ALI group. Similarly, other studies involving respiratory diseases have reported comparable findings. Cavalcanti et al. (2024) (Cavalcanti et al. 2024), Indeed, in a murine model of combined allergic rhinitis and asthma syndrome (CARAS), oral supplementation of animals with *Lacticaseibacillus paracasei* reduced leukocyte infiltration and consequently restored lung architecture. In a comparable manner, Kim et al. (2024) (Kim et al. 2024) demonstrated that oral supplementation with *Lactiplantibacillus plantarum* KF511 (1 × 10⁸ CFU/mL) for 28 days attenuated lung injury in mice with cigarette smoke extract–induced chronic obstructive pulmonary disease (COPD). The strain used inhibited the release of pro-inflammatory cytokines and chemokines, further corroborating the findings of our study.

Lipopolysaccharide (LPS), recognized by the TLR4, triggers a signaling cascade that culminates in the activation of the transcription factor NF-κB, thereby promoting the expression of pro-inflammatory mediators and contributing to the amplification of the pulmonary inflammatory response (Wei et al. 2023). The immunohistochemical analysis for Toll-like receptor 4 and NF-κB revealed that positive staining was noticeably more subtle in the ALI+Lp62 group compared with the ALI group, which still exhibited intense labeling within the cellular infiltrate.

Recent evidence reinforces that probiotic, particularly *Lactobacillus* strains, play a significant modulatory role in airway inflammation through the negative regulation of TLR4-mediated signaling. In murine models of allergic or LPS-induced airway inflammation, strains such as *L. rhamnosus* GG (Lin et al. 2025), *Lactobacillus acidophilus* LA-5 (Wu et al. 2022), *Limosilactobacillus fermentum* (formely *Lactobacillus fermentum*) CECT5716 (Wang et al. ), *L. rhamnosus* GG and *B. longum* (Khailova et al. 2014) have demonstrated the ability to reduce pulmonary TLR4 expression, attenuate downstream MyD88/NF-κB activation, and consequently decrease the production of pro-inflammatory cytokines. These effects were accompanied by reduced cellular infiltration, diminished mucus hypersecretion, and preservation of lung architecture.

Several studies have demonstrated that probiotics, particularly *Lactobacillus* strains, exert robust anti-inflammatory effects in animal models of respiratory diseases through the inhibition of the NF-κB pathway. In murine models of cigarette smoke-induced COPD, for example, *L. rhamnosus* significantly reduced pulmonary NF-κB activation, accompanied by decreased expression of TLR2/TLR4, lower levels of pro-inflammatory cytokines, and reduced neutrophilic infiltration (Mortaz et al. 2015). Similar results were observed with *L. rhamnosus* GG *e B. longum* (Khailova et al. 2014), which, in widely used respiratory-related cellular models, markedly suppressed NF-κB activation and its downstream mediators, reinforcing the role of these microorganisms in controlling inflammatory responses. It is noteworthy that, in an experimental model of medication-related osteonecrosis of the jaws, Lp62 administered established by our research group at the same dosage likewise demonstrated the capacity to attenuate NF-κB activation in osteocytes (Nascimento et al. 2026).

The transcription factor NF-κB plays a central role in the early inflammatory response by inducing the expression of multiple pro-inflammatory mediators. Among these, interleukin-17 (IL-17) contributes to the amplification of acute inflammation through the stimulation of chemokine and cytokine production. Although IL-17 is known to participate in neutrophil-oriented responses, in the present study its evaluation was restricted to immunohistochemical detection in lung tissue, providing a qualitative assessment of local expression rather than quantitative measurement in BALF or serum (Liu et al. 2025).

The immunohistochemical analysis for IL-17 revealed that positive staining was noticeably more subtle in the ALI+Lp62 group compared with the ALI group, which still exhibited intense labeling within the cellular infiltrate.

Our study showed that mice supplemented with Lp62 had lower levels of IL-17 in lung tissue compared to those that did not receive the lactobacillus. Similar findings were observed with *Lactobacillus gasseri* (Jan et al. 2012) and *L. paracasei* HB89 which, in allergen or fine particulate matter respiratory inflammation models, suppressed IL-17 production and thereby contributing to diminished tissue damage and attenuation of bronchial hyperresponsiveness. In a murine model of allergic asthma induced for *Dermatophagoides pteronyssinus*, oral administration of *L. gasseri* 4 times 10^6^ CFU/mL attenuated the influx of inflammatory cells into the airways and reduced TNF‑α and IL‑17 A levels in the bronchoalveolar lavage fluid, indicating a direct modulation of cytokine‑mediated inflammatory pathways. In the Balb/c murine model exposed to fine particulate matter (PM2.5), administration of *L. paracasei* HB89 (2 × 10^9^ CFU) attenuated the induced allergic inflammation by reducing leukocytosis, IgE responses, and pulmonary immune-cell proliferation, while rebalancing the cytokine profile, notably decreasing IL-4, IL-5, IL-13, and IL-17 A (Lin et al. 2020).

The inflammatory process involves extensive communication among immune cells mediated by cytokines. Among these mediators, IL‑1β plays a central role, as its increased levels in the lungs of animals with ALI enhance pulmonary epithelial permeability and contribute to tissue damage and amplification of inflammation (Antunes et al. 2022). Furthermore, IL-1β, along with IL-6, are cytokines involved in the induction of ILC3 and the differentiation of CD4^+^ T cells to the Th17 profile, both of which produce IL-17 A (To et al. 2024). In this contextt Lp62 played an important role in reducing IL1β levels in the BALF of mice subjected to ALI by LPS. Similarly, in a dextran sodium sulfate (DSS)-induced colitis model, *Lactobacillus johnsonii* N5 suppressed colonic pro-inflammatory factors such as TNF, IL-1β, IL-6, and CXCL1, reducing IL-17 A production (Liu et al. 2025).

Taken together, these findings support the notion that negative modulation of the TLR4–NF-κB –IL-17/IL-1β constitutes a central mechanism by which probiotics like Lp62 mitigate pulmonary inflammation, highlighting their potential as adjuvant strategies in the management of inflammatory respiratory diseases and this finding supports previous evidence demonstrating that Lp62 are capable of modulating inflammatory pathways by suppressing or regulating NF-κB activation (Nascimento et al. 2026).

Although the LPS-induced acute lung injury (ALI) model in mice is widely used due to its reproducibility and its ability to mimic the early stages of pulmonary inflammation in humans, it presents important limitations that must be considered when interpreting the results. The inflammatory response triggered by LPS is predominantly mediated by TLR4 activation and is characterized by an acute, abrupt, and highly neutrophilic profile, which does not fully reflect the temporal and cellular complexity observed in clinical forms of ALI, which involve exudative, proliferative, and fibrotic phases (Ciesielska et al. 2021). Moreover, the 24-hour time frame captures only the initial peak of inflammation, preventing the assessment of resolution processes, tissue remodeling, or late consequences of injury (Luo et al. 2025). Another relevant point is that LPS administration represents a single and direct stimulus, differing from multifactorial human etiologies such as sepsis, trauma, or pneumonia, in which multiple immune pathways are activated simultaneously. Thus, although useful for investigating early mechanisms of pulmonary inflammation, this model has limitations regarding its extrapolation to the full pathophysiology of ALI in humans. The exclusive use of male mice represents an additional limitation, as sex‑dependent differences in inflammatory responses were not assessed in this study.

Additional methodological limitations should also be acknowledged. BALF analysis was restricted to total cell counts, without differential quantification of neutrophils, macrophages, lymphocytes, or eosinophils, which limits the ability to characterize specific leukocyte populations involved in the inflammatory response. Likewise, only IL‑1β was quantified among soluble mediators, and no broader cytokine or chemokine panel was assessed, preventing a more comprehensive evaluation of the inflammatory network associated with TLR4/NF‑κB activation. Furthermore, the study did not include systemic or gastrointestinal assessments—such as microbiota composition, intestinal permeability, or circulating metabolites—therefore precluding any inference regarding systemic mechanisms or gut–lung axis involvement. Future studies incorporating differential BALF cytology, expanded cytokine profiling, longer observation periods, and systemic immunological analyses will be essential to deepen the understanding of the biological effects of *Lactiplantibacillus plantarum* 6.2 in ALI.

## Conclusion

Oral supplementation with *Lactiplantibacillus plantarum* 6.2 reduced cellular migration and protein leakage in the bronchoalveolar lavage fluid, decreased pulmonary lesions, and preserved alveolar architecture in the LPS‑induced ALI model. A concomitant reduction in TLR4, NF‑κB and IL‑17 immunostaining was also observed in lung tissue, indicating lower local inflammatory activation. Together, these findings demonstrate that Lp62 exerts a pulmonary anti‑inflammatory effect in this experimental model, although further studies are required to clarify the underlying mechanisms and assess potential systemic effects.

## Data Availability

This is a research paper extracted from a dissertation. Complete data are available upon request from the corresponding author.
